# Dietary effects on body composition, glucose metabolism, and longevity are modulated by skeletal muscle mitochondrial uncoupling in mice

**DOI:** 10.1111/j.1474-9726.2010.00648.x

**Published:** 2011-02

**Authors:** Susanne Keipert, Anja Voigt, Susanne Klaus

**Affiliations:** Group of Energy Metabolism, German Institute of Human NutritionArthur-Scheunert-Allee 114-116, 14558 Nuthetal, Germany

**Keywords:** aging, body composition, energy expenditure, glucose tolerance test, high-fat diet, life expectancy, macronutrients, mouse, physical activity

## Abstract

Little is known about how diet and energy metabolism interact in determination of lifespan under ad libitum feeding. From 12 weeks of age until death, male and female wild-type (WT) and transgenic (TG) mice with increased skeletal muscle mitochondrial uncoupling (HSA-mUCP1 mice) were fed one of three different semisynthetic diets differing in macronutrient ratio: control (high-carbohydrate/low-fat-HCLF) and two high-fat diets: high-carbohydrate/high-fat (HCHF), and low-carbohydrate/high-fat (LCHF). Compared to control and LCHF, HCHF feeding rapidly and significantly increased body fat content in WT. Median lifespan of WT was decreased by 33% (HCHF) and 7% (LCHF) compared to HCLF. HCHF significantly increased insulin resistance (HOMA) of WT from 24 weeks on compared to control. TG mice had lower lean body mass and increased energy expenditure, insulin sensitivity, and maximum lifespan (+10%) compared to WT. They showed a delayed development of obesity on HCHF but reached similar maximum adiposity as WT. TG median lifespan was only slightly reduced by HCHF (−7%) and unaffected by LCHF compared to control. Correlation analyses showed that decreased longevity was more strongly linked to a high rate of fat gain than to adiposity itself. Furthermore, insulin resistance was negatively and weight-specific energy expenditure was positively correlated with longevity. We conclude that (i) dietary macronutrient ratios strongly affected obesity development, glucose homeostasis, and longevity, (ii) that skeletal muscle mitochondrial uncoupling alleviated the detrimental effects of high-fat diets, and (iii) that early imbalances in energy homeostasis leading to increased insulin resistance are predictive for a decreased lifespan.

## Introduction

Obesity rates are increasing dramatically worldwide, which constitutes a serious strain on health care systems because of the obesity-associated health problems such as cardiovascular disease or diabetes (Withrow & Alter, 2010). It is generally accepted that obesity leads to a reduced life expectancy because of these associated health problems. The most important dietary factor contributing to the development of obesity is a high caloric intake, which is linked to a high dietary energy density (Rolls, 2009). Energy density of fat is twice as high as that of protein and carbohydrates, and dietary fat content has long been considered the most important factor contributing to increasing obesity prevalence (Bray *et al.*, 2004). High-fat diets are known to induce obesity in many animal models and have also been shown to reduce lifespan (Otabe *et al.*, 2007). However, the respective roles of dietary carbohydrate and protein in development or prevention of obesity are currently discussed quite intensively. We have shown previously in a mouse model that increasing the protein:carbohydrate ratio in a high-fat diet delayed development of obesity and improved glucose homeostasis (Klaus, 2005). A recent meta-analysis of controlled human trials showed that low-carbohydrate/high-protein diets are more effective at short term than low-fat diets in reducing weight and cardiovascular disease in humans (Hession *et al.*, 2009). It has therefore been suggested that increasing the dietary protein to carbohydrate ratio could be effective in improving weight and metabolic control in humans (Hession *et al.*, 2009; Acheson, 2010). However, there are still not sufficient data on long-term health effects of such diets.

Regarding dietary effects on lifespan, dietary (DR) or caloric restriction (CR) has been the main research focus for several decades. Many effects of DR are thought to be linked to changes in macronutrient balance, but the effects of macronutrient balance on lifespan were almost exclusively investigated in insects so far (Simpson & Raubenheimer, 2009). In *Drosophila* it was shown that the addition of essential amino acids to a lifespan increasing DR regime reduced lifespan to the level of fully fed flies, suggesting an imbalance of dietary amino acid ratios to be a key factor in regulation of lifespan (Grandison *et al.*, 2009). DR is generally considered to increase lifespan in species ranging from yeast over flies and mice to monkeys (Fontana *et al.*, 2010), but recently two independent studies showed that effects of DR on different recombinant inbred mice strains range from life extension to life shortening (Liao *et al.*, 2010; Rikke *et al.*, 2010). On the other hand, very little is known about how dietary macronutrient composition affects lifespan when fed *ad libitum*, which is probably more relevant to the human situation. Particularly, there is a scarcity of studies examining the role of dietary macronutrient ratios on aging and longevity in mammals. One study performed in two different strains of wistar rats did not find conclusive evidence for an influence of different dietary macronutrient contents on longevity (Preuss, 1997). Another study performed in genetically obese and nonobese mice reported a high-fat diet–induced decrease in lifespan in genetically obese mice only (Smith *et al.*, 1991).

It has lately emerged that aging is not a random process but determined by a genetically regulated longevity network which can be modulated genetically and also by environmental factors (Ladiges *et al.*, 2009; Blagosklonny *et al.*, 2010; Chen *et al.*, 2010). But so far, little is known about the interaction of diet composition and genetic and metabolic factors with regard to aging and lifespan. Therefore, here we aimed to dissect the role of macronutrients on obesity, aging and lifespan and their interaction with energy and glucose homeostasis to explore their respective role in aging and longevity. To this means, we investigated the effects of lifelong feeding of three different, semisynthetic diets with identical macronutrient sources but different macronutrient ratios, two of which had an identical high-fat content but different protein:carbohydrate ratios. These diets were fed to wild-type mice (WT) and transgenic mice (TG) with skeletal muscle mitochondrial uncoupling induced by ectopic expression of the uncoupling protein 1 in skeletal muscle (HSA-UCP1 mice) (Klaus *et al.*, 2005). UCP1 expression is normally restricted to brown fat mitochondria where it is responsible for cold-induced nonshivering thermogenesis (Klaus *et al.*, 1991). We found that UCP1 displayed native behavior in isolated skeletal muscle mitochondria from TG mice. Mitochondria showed an increased uncoupling that was activated by fatty acids and could be inhibited by purine nucleotides (Keipert *et al.*, 2010). On the other hand, UCP1 expression in brown fat was not affected by the transgene (Keipert, unpublished results). HSA-UCP1 mice display decreased body weight (lean mass and fat mass), increased energy expenditure, and increased metabolic flexibility (i.e. a faster switch between carbohydrate und lipid oxidation), as well as lower insulin levels and increased insulin sensitivity (Klaus *et al.*, 2005; Katterle *et al.*, 2008; Neschen *et al.*, 2008). Also, using a different transgenic mouse model of UCP1 expression in skeletal muscle, a slightly prolonged median lifespan of about 10% has been shown in transgenic mice on a standard chow diet (Gates *et al.*, 2007). However, we have shown that HSA-UCP1 mice are not in general resistant to diet-induced obesity but rather show a delayed development of obesity and a dissociation of obesity and insulin sensitivity when fed a high-carbohydrate/high-fat diet (Katterle *et al.*, 2008). Therefore, we wanted to investigate whether the increased insulin sensitivity of TG mice could be able to counteract deleterious effects of high-fat diets even in the presence of obesity. Inclusion of this transgenic mouse model can also help to identify individual predictive markers for lifespan by broadening the range of phenotypic variability regarding energy and glucose homeostasis. The aim of our study was thus to examine the role of dietary macronutrient ratios on lifespan and on energy metabolism, obesity development, and glucose homeostasis throughout life. Furthermore, we aimed to investigate whether alterations in skeletal muscle energy metabolism that lead to increased insulin sensitivity are able to counteract possible deleterious dietary effects on longevity and survival. Finally, we tried to identify early factors of energy metabolism and/or glucose homeostasis, which could represent metabolic biomarkers predictive for lifespan and survival.

## Results

### Lifespan

**Fig. 1:**
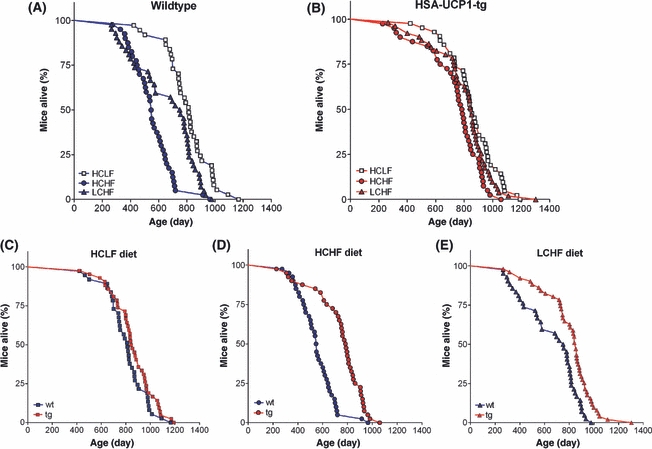
Kaplan–Meier survival curves of wild-type (WT) and transgenic (TG) (HSA-UCP1) mice fed three different semisynthetic macronutrient diets ad libitum from 12 weeks of age (both sexes combined). A significant reduction in lifespan of WT high-carbohydrate/high-fat (HCHF) fed mice compared to high-carbohydrate/low-fat (HCLF) fed mice was observed (A) (*P* < 0.0001, log-rank test). Lifespan of low-carbohydrate/high-fat (LCHF) fed WT mice was significantly different from both other diets. In TG animals (B) the only significant difference in survival was obvious between the control (HCLF) diet and the HCHF diet (*P* < 0.003, log-rank test). (C–E) Lifespan of TG mice in direct comparison to WT mice on each of the three different diets. There was no significant difference in survival between TG and WT on the control HCLF diet (C), but a significantly increased median lifespan of TG on the HCHF diet (+42%) (D) and on the LCHF diet (+13%) (E). *n* = 37–50.

Analyses of survival curves (Mantel-Cox, log-rank test) as well as mean lifespan (Mann–Whitney test) did not show significant gender differences in either WT or TG mice. Also, maximum lifespan (defined as oldest surviving 10% of all mice) was not different between males and females (males 1041 ± 28 days, females 1065 ± 7 days). Therefore, survival data were analyzed for both sexes combined (Fig. S1 and Table S1 for survival characteristics of male and females analyzed separately). There was a significant diet effect on survival of WT mice with mice on the control (HCLF) diet showing the longest survival (Fig. 1A) and the highest mean lifespan (Table 1). Compared to the control diet, mean lifespan was reduced on the two high-fat diets (−17% on the (LCHF diet and −33% on the HCHF diet). Log-rank (Mantel-Cox) test showed significant differences in survival between all three diets in WT (HCLF vs. HCHF *P* < 0.0001; HCLF vs. LCHF *P* = 0.004; HCHF vs. LCHF *P* = 0.003).

**Table 1:** Survival characteristics (days) of wild-type (WT) and transgenic (HSA-UCP1) mice fed three different semisynthetic macronutrient diets (both sexes combined)

	Diet	Median	Mean	Range	Oldest 10%	Youngest 10%	*n*
WT	HCLF	814	807 ± 27	423–1167	1066 ± 40	510 ± 49	37
	LCHF	754	668 ± 33	263–976	933 ± 16	295 ± 12	41
	HCHF	548	555 ± 23	273–965	828 ± 65	330 ± 21	42
HSA-UCP1	HCLF	849	857 ± 26	421–1192	1132 ± 29	536 ± 46	42
	LCHF	852	815 ± 27	315–1303	1103 ± 52	420 ± 33	50
	HCHF	785.5	742 ± 31	227–1059	985 ± 27	302 ± 26	40

Oldest (youngest) 10% represents the mean lifespan of the oldest (youngest) surviving 10% of mice from their respective group. Data are reported ± SEM where appropriate.

HCHF, high-carbohydrate/high-fat; HCLF, high-carbohydrate/low-fat; LCHF, low-carbohydrate/high-fat.

In TG mice survival was much less affected by the diets than in WT (Fig 1B). The only significant difference in survival was obvious between the control (HCLF) diet and the HCHF diet (*P* < 0.003, Mantel-Cox test). Mean lifespan of TG on the HCHF diet was reduced by 13% compared to the control (HCLF) diet (Table 1).

Overall, in TG mice maximum lifespan (defined as the oldest surviving 10% of all mice of one genotype) was increased by about 100 days compared to WT (WT 992 ± 20 days, TG 1094 ± 22 days, *P* = 0.002). Minimum lifespan was increased as well by over 20% (youngest 10% surviving mice in WT 335 ± 12 days, in TG 411 ± 27 days, *P* < 0.05), indicating an overall shift in early mortality. Comparison of TG with WT mice on the different diets showed significantly different survival curves of TG on the two high-fat diets (HCHF, *P* < 0.0001; LCHF, *P* < 0.01, Mantel-Cox test) compared to WT, but not on the HCLF control diet (Fig. 1C–E). Mean lifespan of WT and TG was similar on the control (HCLF) diet but not on the two high-fat diets. Compared to WT, mean lifespan of TG mice on the high-fat diets was significantly increased by 22% (LCHF) and 34% (HCHF) (Table 1).

### Body composition

**Fig. 2:**
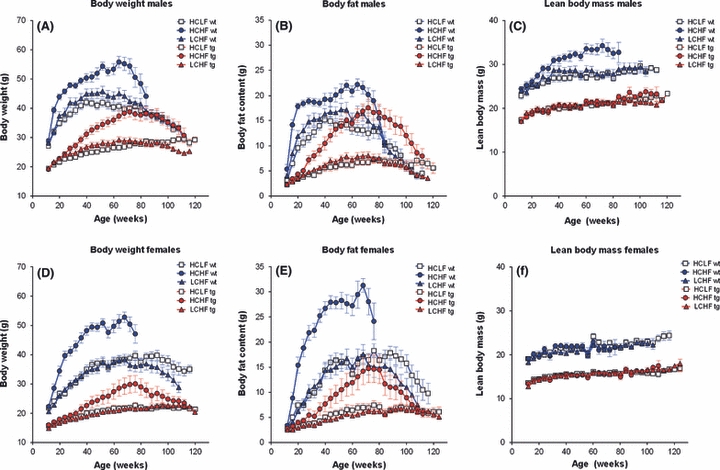
Body weight and body composition development of wild-type (WT) and transgenic (TG) (HSA-UCP1) mice fed three different semisynthetic macronutrient diets ad libitum from 12 weeks of age. Body weight (A, D), body fat content (B, E), and lean body mass (C, F) of male (A–C) and female (D–F) mice. Data are plotted until the week of 50% survival (initial *n* = 17–27). Body weight and body fat was significantly increased in WT high-carbohydrate/high-fat (HCHF) compared to high-carbohydrate/low-fat (HCLF) mice from week 16 on, in TG HCHF compared to HCLF mice from week 36 on.

Body weight and body composition throughout life are presented in Fig. 2. Starting body weight of TG was lower than that of WT, mainly attributed not only to decreased lean body mass (LBM) but also to a slightly lower body fat content (Fig. 2, Table 2). At this time (week 12), relative body fat content of TG mice was slightly decreased in males and slightly increased in females compared to WT (data not shown). Feeding of the HCHF diet increased body weight and body fat significantly from week 16 on compared to the control diet (HCLF) in both male and female WT mice. In contrast, compared to the control (HCLF) diet, the LCHF diet led to only minimal increases in body weight and body fat in male mice (Fig. 2A,B) and even slightly decreased body weight and body fat in female mice after 70 weeks of age (Fig. 2D,E).

**Table 2:** Phenotypic data of male and female wild-type (WT) and transgenic (TG) (HSA-UCP1) mice fed three different semisynthetic macronutrient diets ad libitum from 12 weeks of age (data are shown as mean ± SEM)

	WT			HSA-UCP1 (TG)			anova	
			
	HCLF	HCHF	LCHF	HCLF	HCHF	LCHF	Diet	Genotype
Males								
Before dietary switch								
Body weight week 12 (g)	27.1 ± 0.7	28.8 ± 0.8	28.9 ± 0.6	19.4 ± 0.6	19.1 ± 0.4	19.3 ± 0.5	ns	<0.0001
Body fat mass week 12 (g)	3.0 ± 0.2^a^	4.1 ± 1^b^	4.2 ± 0.4^b^	2.2 ± 0.1	2.3 ± 0.1	2.3 ± 0.1	<0.05	<0.0001
Lean body mass week 12 (g)	24.1 ± 0.6	24.3 ± 0.6	24.7 ± 0.4	17.1 ± 0.6	16.8 ± 0.4	17.0 ± 0.4	ns	<0.0001
After dietary switch								
Body weight week 24 (g)	36.2 ± 1.1^a^	45.4 ± 0.9^b^	41.7 ± 1.3^ab^	22.6 ± 0.6	24.3 ± 0.8	23.9 ± 0.7	<0.0001	<0.0001
Body fat mass week 24 (g)	10.8 ± 0.7^a^	18.2 ± 0.7^b^	14.2 ± 1.1^c^	3.5 ± 0.2	5.0 ± 0.4	4.2 ± 0.3	<0.0001	<0.0001
Time of max BF (week)	53 ± 4	53 ± 3	48 ± 2	75 ± 6^a^	72 ± 4^ab^	60 ± 4^b^	<0.05	<0.0001
Max body fat (g)	16.9 ± 0.9^a^	23.0 ± 0.9^b^	18.9 ± 1.1^a^	8.4 ± 0.8^a^	18.8 ± 1.5^b^	9.4 ± 0.8^a^	<0.0001	<0.0001
Physical activity week 31 (counts/day/1000)	410 ± 35	352 ± 53	327 ± 40	435 ± 57	452 ± 53	493 ± 46	ns	<0.05
Females								
Before dietary switch								
Body weight week 12 (g)	22.0 ± 0.3	22.3 ± 0.7	20.7 ± 0.4	15.8 ± 0.4	16.1 ± 0.3	14.9 ± 0.3	<0.01	<0.0001
Lean body mass week 12 (g)	19.1 ± 0.3	19.1 ± 0.4	18.3 ± 0.3	13.6 ± 0.3	13.4 ± 0.2	12.8 ± 0.3	<0.05	<0.0001
Body fat mass week 12 (g)	2.8 ± 0.2	3.0 ± 0,4	2.4 ± 0.1	2.2 ± 0.1	2.4 ± 0.2	2.1 ± 0.1	ns	<0.01
After dietary switch								
Body weight week 24 (g)	28.1 ± 0.8^a^	39.7 ± 1.6^b^	28.1 ± 0.9^a^	17.9 ± 0.5	18.7 ± 0.6	17.4 ± 0.3	<0.0001	<0.0001
Body fat mass week 24 (g)	7.6 ± 1.0^a^	18.4 ± 1.8^b^	7.5 ± 0.8^a^	3.0 ± 0.2	4.0 ± 0.5	2.9 ± 0.2	<0.0001	<0.0001
Time of max BF (week)	81 ± 5^a^	59 ± 3^b^	56 ± 4^b^	95 ± 5^a^	76 ± 6^b^	86 ± 5^ab^	<0.0001	<0.0001
Max body fat (g)	20.5 ± 1.4^a^	31.3 ± 1.6^b^	17.5 ± 1.9^a^	8.2 ± 0.8^a^	15.5 ± 2.6^b^	7.2 ± 0.6^a^	<0.0001	<0.0001
Physical activity week 31 (counts/day/1000)	685 ± 106	555 ± 69	765 ± 75	479 ± 57	578 ± 112	767 ± 67	<0.05	ns

Different superscript letters denote significant differences between the diet groups within one genotype (anova).

HCLF, high-carbohydrate/low-fat; HCHF, high-carbohydrate/high-fat; LCHF, low-carbohydrate/high-fat.

Lean body mass (LBM) was always lower in TG mice than in WT. In both, it showed only minor increases until about 30 weeks of age and stayed rather stable thereafter. Diet composition did not affect LBM at all in female WT and TG (Fig. 2F). Only male WT mice showed an increase in LBM on the HCHF diet compared to the other two diets from about week 30 on (Fig. 2C).

**Fig. 3:**
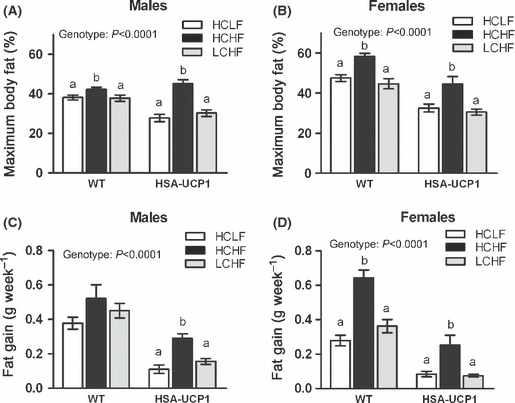
Maximum body fat content and rate of body fat gain of wild-type (WT) and transgenic (TG) (HSA-UCP1) mice fed three different semisynthetic macronutrient diets ad libitum from 12 weeks of age. Maximum relative body fat levels (A, B) and rate of fat gain from week 12 until maximum body fat levels were reached (C, D) of male (A, C) and female (B, D) mice. Different superscript letters denote significant differences between the diet groups within one genotype (anova, *n* = 17–26).

Interestingly, in HCHF fed mice, body fat reached a plateau in WT male mice at about 20 weeks but was further increased with age in female WT mice which reached plateau after about 50 weeks of age. The time point at which mice reached their maximum body weight and body fat levels was highly influenced by genotype and diet in both male and female mice (Table 2). Because of the apparent differences in maximum body fat and the velocity of body fat gain, we also calculated maximum body fat levels and the rate of fat gain from dietary intervention start (week 12) until maximum body fat was reached (Fig. 3). Maximum relative body fat levels were not different between the control (HCLF) diet and LCHF diet in either sex or genotype (Fig. 3A,B), which shows that increasing the protein content in a high-fat diet regime completely prevented the development of high-fat-induced obesity. On the other hand, feeding of the HCHF diet resulted in increased maximum body fat levels compared to the other two diets in WT and TG males and females (Fig. 3A,B). Interestingly, male TG mice reached similar maximum body fat levels as WT (Fig. 3A), confirming our previous findings that TG mice are not generally protected against the development of obesity (Katterle *et al.*, 2008). The rates of body weight and body fat gain were highly correlated (*R* = 0.96). Body fat variability thus explained over 90% of body weight variability, which clearly shows that it is the most important contributor to body weight changes in adult mice. The rate of body fat gain was lowest in male and female TG mice on the control (HCLF) diet and highest in female WT mice on the HCHF diet, followed by males on the HCHF diet (Fig. 3D,E). Overall, TG mice showed much lower velocity of body fat accretion than WT mice.

### Energy expenditure and physical activity

**Fig. 4:**
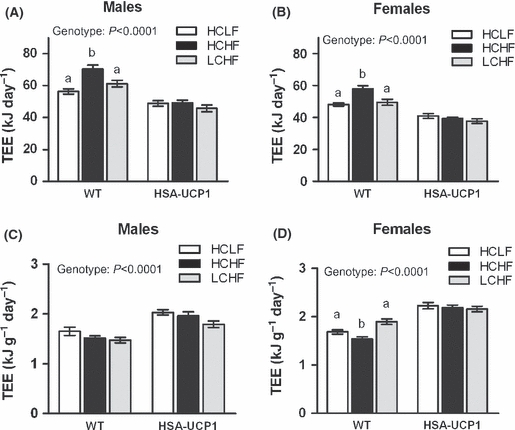
Total daily energy expenditure (TEE) of wild-type (WT) and transgenic (TG) (HSA-UCP1) mice fed three different semisynthetic macronutrient diets ad libitum from 12 weeks of age. TEE per animal (A, B) and weight-specific TEE (C, D) of male (A, C) and female (B, D) mice was measured at 31 weeks of age. Different superscript letters denote significant differences between the diet groups within one genotype (anova, *n* = 8–13).

Energy expenditure was measured by indirect calorimetry continuously over 24 h in a subset of mice in week 31. Total energy expenditure was higher in WT mice than in TG mice (Fig. 4A,B) because of the higher body weight of WT. The increased body weight also resulted in an increased total daily energy expenditure (TEE) of both male and female WT mice on the HCHF diet compared to the other two diets. TEE was determined by body weight to almost 80% (*r* = 0.885). When corrected for body weight, weight-specific TEE was significantly affected by genotype and diet in both males and females and increased in TG compared to WT (Fig 4C,D) as reported previously (Klaus *et al.*, 2005; Katterle *et al.*, 2008). However, post hoc tests only showed significant differences in weight-specific TEE of female WT on the HCHF diet compared to the other two diets (Fig. 4D). Together this suggests that diet composition by itself had only minor, if any effects on energy expenditure. Similar results were seen when resting energy expenditure was calculated (data not shown).

Spontaneous physical activity was measured over a period of 3 days after the measurement of energy expenditure by using infrared movement sensors. As shown in Table 2, in male mice there was a small genotype effect with TG mice showing slightly higher activity levels than WT with no significant influence of diet. Female mice on the other hand showed overall higher activity levels than males and minor diet effects with a tendency for increased activity levels on the LCHF diet.

### Glucose homeostasis and insulin resistance

**Fig. 5:**
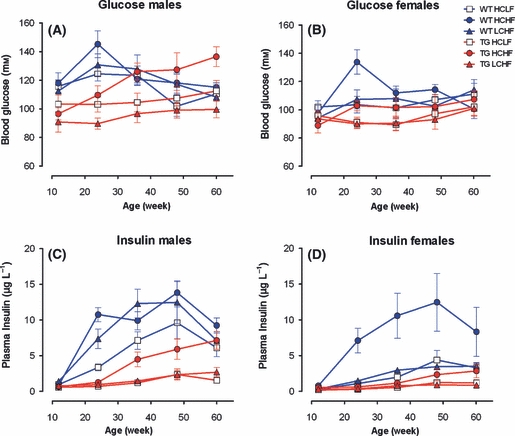
Development of blood glucose and insulin levels (3 h fasted) in wild-type (WT) and transgenic (TG) (HSA-UCP1) mice fed three different semisynthetic macronutrient diets ad libitum from 12 weeks of age. (A,B) Blood glucose levels at 24 weeks were significantly influenced by genotype (*P* < 0.0001) and diet (*P* < 0.05) in male (A) and female mice (B) mice (C,D) Plasma insulin was significantly influenced by genotype and diet in male (C) and female mice (D) mice (2-way ANOVA, *n* = 8–13).

Nonfasted (postprandial) blood glucose and insulin levels were measured in a subset of mice in regular intervals from week 12 to 60 of life as presented in Fig. 5. Regardless of the diet, mice were able to retain normo-glycemia throughout this period, which is in general assumed at 60–130 mg dL^−1^ in mice. However, repeated measures analysis showed a significant time effect on blood glucose in male and female mice. In WT mice, there was a tendency for a transient increase in blood glucose at week 24 (i.e. 12 weeks after diet start) on HCHF compared to the other diets and a normalization thereafter (Fig. 5A,B). Blood glucose levels at week 24 were significantly affected by genotype (*P* < 0.0001 in both males and females) and also by diet (*P* < 0.05 in males and < 0.001 in females, Table 3). Female WT mice on HCHF also displayed increased blood glucose in week 24 compared to the other two diets. Before diet start and after week 24, there were no significant genotype effects on blood glucose levels (Table 3). Female TG mice showed rather stable glucose levels until week 60 of the diet (Fig. 5B). Male TG showed a gradual increase in blood glucose on the HCHF diet until week 60 (Fig. 5A, Table 3). Calculation of the area under the curve (AUC) of glucose development during aging showed a significant genotype effect (*P* = 0.002 males, *P* = 0.003 females) but a significant diet effect only in males (*P* = 0.025, two-way anova; Table S2). More pronounced effects of diet and genotype were apparent on the development of insulin levels during aging. Repeated measures analysis showed a highly significant time effect on insulin levels in both male and female (*P* < 0.0001). Insulin increased with age especially in male WT mice, which showed progressively increased insulin levels until about 48 weeks of age with only minor diet effects (Fig. 5C). Female WT mice showed also a similar increase in insulin on the HCHF diet but much lower levels on the other two diets (Fig. 5D, Table 3). AUC for insulin during aging showed highly significant effects of genotype (*P* < 0.0001 for both males and females) and diet (*P* < 0.0001 males, *P* < 0.001 females, two-way anova, Table S2).

**Table 3:** Glucose, Insulin and Homeostasis Model Assessment for insulin resistance (HOMA-IR) at different time points of male and female wild-type (WT) and transgenic (TG) (HSA-UCP1) mice fed three different semisynthetic macronutrient diets ad libitum from 12 weeks of age (data are shown as mean ± SEM)

	WT			HSA-UCP1 (TG)			anova	
			
	HCLF	HCHF	LCHF	HCLF	HCHF	LCHF	Diet	Genotype
Males								
Before dietary switch								
Glucose week 12 (mg dL^−1^)	116 ± 4	118 ± 7	112 ± 8	103 ± 6	96 ± 4	91 ± 7	ns	<0.001
After dietary switch								
Insulin week 24 (μg L^−1^)	3.39 ± 0.48^a^	10.78 ± 1.00^b^	7.51 ± 1.54^b^	0.75 ± 0.12	1.40 ± 0.28	0.95 ± 0.13	<0.0001	<0.0001
Insulin week 60 (μg L^−1^)	6.14 ± 1.22	9.26 ± 1.08	7.16 ± 1.21	1.59 ± 0.31^a^	7.84 ± 1.18^b^	2.60 ± 0.60^a^	<0.0001	<0.0001
Glucose week 24 (mg dL^−1^)	124 ± 5	145 ± 9	131 ± 8	103 ± 5^ab^	110 ± 6^a^	90 ± 4^b^	<0.05	<0.0001
Glucose week 60 (mg dL^−1^)	110 ± 5	115 ± 3	108 ± 8	112 ± 7^ab^	136 ± 7^a^	99 ± 6^b^	<0.01	ns
HOMA-IR week 24	24.9 ± 3.6	92.9 ± 9.2	50.1 ± 8.5	4.7 ± 1.0	9.2 ± 2.1	4.8 ± 0.6	<0.0001	<0.0001
HOMA-IR week 60	40.1 ± 8.1	64.0 ± 7.5	46.2 ± 8.7	10.7 ± 2.1	63.4 ± 9.3	15.0 ± 3.3	<0.0001	0.0002
Females								
Before dietary switch								
Glucose week 12 (mg dL^−1^)	102 ± 4	97 ± 6	94 ± 6	96 ± 4	89 ± 5	93 ± 5	ns	ns
After dietary switch								
Insulin week 24 (μg L^−1^)	1.13 ± 0.36^a^	7.12 ± 1.73^b^	1.50 ± 0.22^a^	0.35 ± 0.06^a^	0.66 ± 0.11^b^	0.40 ± 0.06^ab^	<0.0001	<0.0001
Insulin week 60 (μg L^−1^)	3.34 ± 0.69	8.35 ± 3.41	3.54 ± 0.43	1.24 ± 0.42	2.89 ± 0.95	1.03 ± 0.31	<0.05	<0.01
Glucose week 24 (mg dL^−1^)	104 ± 6^a^	134 ± 9^b^	107 ± 7^a^	91 ± 4	103 ± 4	90 ± 3	<0.001	<0.0001
Glucose week 60 (mg dL^−1^)	111 ± 8	100 ± 7	114 ± 7	102 ± 6	107 ± 6	101 ± 5	ns	ns
HOMA-IR week 24	7.5 ± 2.5	64.4 ± 17.6	9.3 ± 1.1	1.8 ± 0.3	4.1 ± 0.7	2.1 ± 0.3	<0.0001	<0.0001
HOMA-IR week 60	22.3 ± 5.5	58.3 ± 21.8	24.4 ± 3.6	8.5 ± 3.3	18.6 ± 5.6	6.2 ± 2.1	0.021	0.002

Different superscript letters denote significant differences between the diet groups within one genotype (anova).

HCLF, high-carbohydrate/low-fat; HCHF, high-carbohydrate/high-fat; LCHF, low-carbohydrate/high-fat.

Homeostasis Model Assessment for insulin resistance (HOMA-IR) was calculated as a correlate for insulin sensitivity according to Lansang *et al.*, (2001). Similar to insulin data, there were highly significant effects of genotype and diet in both males and females (Table 3). Already, at week 24 WT mice on the HCHF diet showed significantly increased HOMA-IR compared to the control (HCLF) but also compared to the LCHF diet, which was most pronounced in male mice. At week 60, HOMA-IR of WT mice was not further increased in HCHF, indicating that maximum levels of insulin resistance had already been reached after 12 weeks of dietary intervention. TG mice showed lower HOMA-IR values than WT at both time points. However, at week 60, insulin resistance was similar in male TG and WT mice fed the HCHF diet. In female TG mice, the diet effect was less pronounced than in males (Table 3).

**Fig. 6:**
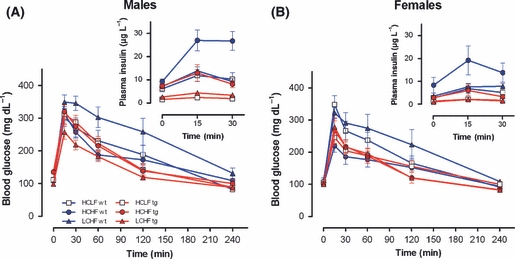
Glucose tolerance test performed at 60 weeks of age in wild-type (WT) and transgenic (TG) (HSA-UCP1) mice fed three different semisynthetic macronutrient diets ad libitum from 12 weeks of age. Blood glucose levels in male (A) and female (B) mice until 240 min after glucose injection (*n* = 7–13). Glucose (2 mg g^−1^) was applied i.p. to 3-h fasted mice (*n* = 7–13). The inserts show corresponding insulin levels before, 15, and 30 min after glucose application.

At 60 weeks of age, a glucose tolerance test (GTT) was performed (Fig. 6) with measurements of blood glucose until 240 min after glucose injection and determination of plasma insulin at baseline, 15, and 30 min after glucose injection (Fig 6, inserts). There was a significant effect of genotype (*P* < 0.01 and *P* < 0.05 in male and female mice, respectively, two-way anova) but not of diet on AUC of blood glucose. Insulin AUC of male mice was significantly affected by genotype and diet (*P* < 0.0001 for both, two-way anova) with the HCHF group significantly increased in comparison with the other two diets in both WT and TG mice. In female mice, AUC of insulin was significantly affected by genotype (*P* < 0.001) and diet (*P* < 0.01, two-way anova, Table S2).

### Correlations of phenotypic markers with lifespan

To identify the metabolic traits that are predictive for increased or decreased lifespan, we performed a correlation analysis between lifespan of individual mice and different phenotypic markers as shown in Table 4. In TG but not in WT mice, body weight before the start of the dietary intervention (week 12) was negatively correlated with lifespan. However, as early as 12 weeks after begin of the dietary intervention (week 24), body weight of both WT and TG mice showed a significant negative correlation with lifespan, which was even stronger in both genotypes combined. This was mainly linked to body fat content, which showed a very similar negative correlation to lifespan. Interestingly, maximum body fat levels were less good predictors of lifespan, suggesting that early increases in body fat are more deleterious than the maximum fatness reached in life. Also, the time at which maximum body fat was reached showed the strongest correlation (*r* = 0.605, *P* < 0.0001, WT and TG combined) with lifespan of all measured parameters. However, this time variable is not independent from lifespan, which is also a time variable (mice that live longer have more time to accumulate body fat). Therefore, we calculated the rate of body fat gain (as described above), which proved to be a strong predictor of life expectancy explaining about 23% of lifespan variation in WT mice (Table 4). This is strongly supported by data in TG mice, which showed a similar correlation with the rate of body fat gain. There was no significant difference in the respective regression curves between WT and TG although TG mice are significantly smaller (Katterle *et al.*, 2008) and lighter than WT. In WT and TG combined, the rate of fat gain explained over 30% of the variation in life expectancy (*r*^2^ = 0.31, *P* < 0.0001).

**Table 4:** Spearman correlation coefficients of selected phenotypic markers with life expectancy of wild-type (WT) and transgenic HSA-UCP1 (TG) mice fed three different semisynthetic macronutrient diets. Male, female and diet groups were combined

	Correlation coefficient		
	WT (*n*)	TG (*n*)	WT+TG (*n*)
Body weight week 12 (g)	ns (115)	−0.419*** (125)	−0.423*** (240)
Body weight week 24 (g)	−0.397*** (114)	−0.451*** (125)	−0.506*** (239)
Body fat week 24 (g)	−0.338*** (114)	−0.386***(125)	−0.471*** (239)
Maximum body fat (g)	−0.197* (115)	−0.238* (125)	−0.373*** (240)
Time of maximum body fat (week)	0.427*** (115)	0.658*** (125)	0.605*** (240)
Rate of fat gain (g per week)	−0.475*** (115)	−0.501*** (125)	−0.561*** (240)
TEE (kJ per day)	−0.406** (62)**	−0.391** (61)	−0.520*** (123)
Weight-specific TEE (kJ/d*g BW)	ns (62)	0.232* (61)	0.416*** (123)
Physical activity (wk31)	ns (62)	ns (56)	ns (118)
Glucose week 12 (mg dL^−1^)	ns (48)	ns (61)	ns (109)
Glucose week 24 (mg dL^−1^)	−0.292* (54)	ns (66)	−0.329*** (120)
Insulin week 24 (μg L^−1^)	ns (52)	ns (66)	−0.282*** (118)
HOMA-IR week 24	−0.316* (52)	ns (66)	−0.315*** (118)
Glucose week 60 (mg dL^−1^)	ns (52)	ns (62)	ns (114)
Insulin week 60 (μg L^−1^)	ns (52)	ns (61)	−0.310** (114)
HOMA-IR week 60	ns (52)	−0.270* (59)	−0.313*** (111)

Rate of fat gain corresponds to the weekly increase in body fat from week 12 to the time of maximum body weight. Animal numbers are in brackets.

TEE, total energy expenditure; HOMA-IR, Homeostasis Model Assessment for insulin resistance.

* *P* < 0.05

** *P* < 0.01

*** *P* < 0.001; ns: nonsignificant.

Besides energy intake, energy expenditure can also contribute to body weight gain. TEE per animal (measured in week 31) showed a negative correlation with lifespan. However, as pointed out before, total TEE is to over 80% determined by body weight, which by itself is negatively correlated to lifespan. Therefore, we also calculated weight-specific daily TEE, which showed a very strong negative correlation with both body weight and body fat gain from week 12 to 40 (*R* = −0.794 and −0.715, respectively, *P* < 0.0001, WT and TG combined). Furthermore, weight-specific TEE showed a less strong, but still highly significant correlation with lifespan (*R* = 0.416, *P* < 0.0001) in WT and TG combined. Physical activity levels on the other hand did not show any correlation with lifespan in either WT or TG mice (Table 4).

Not only early changes in energy metabolism, but also in glucose homeostasis are apparently predictive of aging and lifespan. Blood glucose at week 24 but not at later time points showed a significant negative correlation with lifespan (*R* = −0.329, *P* < 0.001; WT and TG together). On the other hand, insulin levels from 24 weeks of age on showed a significant negative correlation with lifespan in WT and TG combined, indicating that increased insulin levels throughout life are correlated with a decreased lifespan. In this context, it is important to note that body fat content in week 24 was very strongly correlated with insulin levels at the same time point (*R* = 0.835, *P* < 0.0001, TG and WT combined). Body fat content thus explains 70% of the variation in insulin levels, but only 40% of the variation in blood glucose (*R* = 0.635, *P* < 0.0001, TG and WT combined). HOMA-IR as a surrogate for insulin resistance also showed a significant negative correlation with lifespan from 24 weeks on, suggesting that early development of insulin resistance is predictive of a reduced lifespan (Table 4).

## Discussion

It is well recognized that aging and longevity are complex characteristics, which are determined by an interplay of genes and environment, with nutrition certainly being one of the most important environmental factors. Recent advances in mouse genetics and the generation of a vast number of genetically modified mouse strains have let to the identification of a number of genes and pathways linked to aging and lifespan. Especially mutant mice with increased longevity are currently investigated to elucidate mechanisms of aging and longevity (Ladiges *et al.*, 2009; Chen *et al.*, 2010). Genetic manipulations in mice leading to alterations in energy metabolism which in turn affect lifespan or aging are suggestive of an important role of metabolic status in the regulation of aging and lifespan (Gates *et al.*, 2007; Wenz *et al.*, 2009; Tocchetti *et al.*, 2010). On the other hand, only little is known about the effects of diet composition on lifespan in mice and even less about the interaction of dietary macronutrient composition and genetic factors with regard to aging and longevity. Here, we show (i) that dietary macronutrient ratios strongly affect obesity development, glucose homeostasis, and longevity in mice, (ii) that skeletal muscle mitochondrial uncoupling alleviates the detrimental effects of high-fat diets on metabolic status and longevity, and (iii) that an early, rapid body fat accretion leading to increased insulin resistance is predictive for a decreased lifespan.

### Macronutrient ratios strongly affect longevity, body composition, and insulin sensitivity

The diet composition was chosen to be able to dissect the specific roles of carbohydrates, protein, and fat, respectively. Although we did not include a chow fed control group, we assume that feeding of our semisynthetic diets did not lead to a reduced lifespan. The median lifespan of WT mice on the control (HCLF) diet was 798 and 825 days for males and females, respectively, and thus well within the range reported for C57BL/6J mice (866 days males, 782 days females) and markedly higher than that reported for CBA/J mice (679 days males, 644 days females) fed a standard low-fat chow diet and kept in a specific pathogen-free environment (Yuan *et al.*, 2009). Thus, we are confident that lifelong feeding of semisynthetic diets supplemented with essential nutrients has no adverse effects on aging and is suitable for use in longevity studies. We analyzed the lifespan in mice of a mixed C57BL/6-CBA background that were inbred for several years. It has been suggested that lifespan studies should be conducted on mice with a uniform genetic background and that a uniform hybrid strain background (as used here) might help minimize idiosyncratic problems associated with some inbred strains (Ladiges *et al.*, 2009).

Concerning dietary effects on WT mice, the survival data clearly show that a diet high in fat and carbohydrates (HCHF) is more deleterious to life expectancy than a diet with the same fat content but decreased carbohydrate and thus increased protein content (LCHF). This argues against the hypothesis that a high protein to nonprotein energy ratio reduces lifespan but underscores the idea that the dietary macronutrient balance rather than the absolute macronutrient intake affect lifespan predominantly (Simpson & Raubenheimer, 2009). The reduced detrimental effect of the LCHF diet on longevity is very strongly linked to the differential effect of the two high-fat diets on adiposity development. Although the LCHF diet contained over 40 energy% of fat, it did not lead to higher maximum adiposity levels than the control HCLF diet with only 17 energy% of fat. The lower body fat gain on the LCHF diet compared to the HCHF diet is probably because of a decreased hyperphagia when protein content of a high-fat diet is increased, which could be related to the known satiating effects of protein (Westerterp-Plantenga *et al.*, 2009). Although we did not measure food intake, our previous data using the same diet composition showed that differences in body weight gain in WT and TG mice on the different diets were predominantly dependent on energy intake. TG mice were found to show a lower energy intake than WT per animal but an increased weight-specific energy intake compared to WT (Katterle *et al.*, 2008).

To our knowledge, our study is the first showing the individual development of body composition throughout life and its dependence on diet composition in mice. Body composition measurements of BL6 mice on a standard chow diet until 2 years of age showed very similar development of body composition as our mice on the control (HCLF) diet, i.e. a steady increase in body fat until over 1 year of age and a decrease in fat mass starting between 80 and 90 weeks of age (Berryman *et al.*, 2010). Furthermore, similar to our study, Berryman *et al.* (2010) found LBM to be rather stable from about 20 weeks of age on. Interestingly, although mice on the control diet (HCLF) showed a gradual decline in body weight from about 40 and 80 weeks on in males and females, respectively, this was exclusively owing to a loss of body fat and not of lean mass. Lean mass was not influenced by diet except in male WT mice on the HCHF diet, which had about 5 g more lean weight after about 60 weeks of age compared to the other groups, the reason for this not being clear yet.

Obesity is associated with disturbances in glucose homeostasis, which can result in the development of type 2 diabetes. Therefore, we assessed glucose homeostasis by regular measurements of blood glucose and insulin, calculation of HOMA-IR, and by performing a glucose tolerance test at 60 weeks of age. The results show clearly that the hybrid mouse strain used here is not very susceptible to the development of diabetes because individual blood glucose levels in 2- to 3-h fasted mice never exceeded 200 mg dL^−1^ until 60 weeks of age and were thus well below the overt diabetes threshold of glucose, which is considered 20 mm (360 mg dL^−1^) for mice (Plum *et al.*, 2000). Nevertheless, insulin sensitivity decreased with age as evident from HOMA-IR. Insulin resistance was consistently higher in males than in females, which is in accordance with the human situation with women considered to be more insulin sensitive than men (Geer & Shen, 2009). Remarkably, there were no diet effects on glucose tolerance in week 60. The ability of HCHF fed mice to maintain normal glucose levels during GTT was obviously attributed to increased basal insulin levels and a fast increase in insulin secretion upon glucose stimulation which compensated for the increased insulin resistance of the HCHF mice. This shows that a habitual high carbohydrate intake led to an adaptive increase in insulin production capacity. Using similar macronutrient diets, we have shown previously that increasing the protein:carbohydrate ratio in a high-fat diet delayed the development of adiposity and improved glucose homeostasis in mice after 10 weeks of ad libitum feeding) (Klaus, 2005). It is now widely accepted that the insulin/IGF-1 pathway plays a prominent role in aging and life expectancy (Bartke, 2008; Narasimhan *et al.*, 2009; Blagosklonny *et al.*, 2010) and many genetic models of increased longevity have mutations in genes of this pathway such as, Igf-1 receptor +/− mice, fat-specific insulin receptor knockout mice, and Irs-1 knockout mice (reviewed in Chen *et al.*, 2010; Ladiges *et al.*, 2009).

### Skeletal muscle mitochondrial uncoupling alleviates detrimental effects of high-fat diets

Altogether our data show that increased muscle mitochondrial uncoupling can alleviate most of the deleterious effects of high-fat diets on aging and survival. Using a different transgenic mouse line with ectopic expression of UCP1 in skeletal muscle (driven by a different promoter than used in our model), Gates and coworkers (2007) showed that these mice had an increased median lifespan of about 10% when kept on a standard chow diet although they did not find a significant increase in maximum lifespan. Lifespan on different diets was not investigated. Our results suggest that the positive metabolic effects of ectopic UCP1 expression in skeletal muscle are highly reproducible. Pooling of mice of all diet groups and both genders revealed a shift toward later mortality in HSA-UCP1 mice. Even more remarkably, TG mice proved to be mostly resistant to the deleterious effect of high-fat diets on longevity HCHF feeding decreased median lifespan of WT by 33% but that of TG mice by only 7% compared to the control diet. Similar results were observed with regard to glucose homeostasis. Insulin sensitivity was much higher in TG than in WT mice as evident from insulin levels and HOMA-IR. A hallmark of mice with ectopic expression of UCP1 in skeletal muscle is indeed the increased insulin sensitivity accompanied by decreased insulin levels, especially under high-fat diet conditions that was observed in our HSA-UCP1 mice, as well as in the transgenic mouse line used by Gates and coworkers (Li *et al.*, 2000; Klaus *et al.*, 2005; Katterle *et al.*, 2008; Neschen *et al.*, 2008), and which was still evident on a genetically obese background (Bernal-Mizrachi *et al.*, 2002). Using insulin tolerance test and hyper insulinemic-euglycemic clamps, we have previously shown increased an insulin sensitivity of HSA-UCP1 mice on chow and high-fat diets compared to WT littermates (Katterle *et al.*, 2008; Neschen *et al.*, 2008). Here, we show additionally that these positive effects seem to persist throughout aging and are therefore most probably linked to the increased survival of HSA-UCP1 mice on high-fat diets.

It is interesting to note that TG mice are of overall smaller body size than wild-type (Klaus *et al.*, 2005; Katterle *et al.*, 2008). Small body size is a striking characteristic of most long-lived mouse mutants such as the Ames Dwarf, Snell Dwarf and Little mouse (reviewed in Bartke, 2005). However, it is assumed that small body size *per se* is not causative for delayed aging, but rather a phenotypic marker of developmental or metabolic characteristics that predispose to increased life expectancy (Bartke, 2005). It has recently been shown that early life growth hormone (GH) treatment of GH-deficient long-lived Ames dwarf mice reduced their lifespan to that of control mice although it failed to completely restore their body weight to control levels (Panici *et al.*, 2010).

The reason for the increased longevity of mice with skeletal muscle uncoupling is not yet clear. It could be attributed to the increased mitochondrial uncoupling itself, which has been suggested to increase longevity by preventing the formation of ROS and was termed the ‘uncoupling to survive’ hypothesis (Brand, 2000). It was reported that individual outbred mice with high metabolic rates showed higher mitochondrial uncoupling and survived longest (Speakman *et al.*, 2004), thus linking high rates of energy expenditure to increased survival. This seems to be confirmed by our data showing increased weight-specific energy expenditure in TG mice irrespective of the diet. However, it should be noted that normalization of energy expenditure to body weight might not be ideal when comparing mice with largely different LBM and fat mass (FM). Regression-based approaches that account for variation in both FM and LBM might be better for normalization of energy expenditure in mice (Kaiyala *et al.*, 2010).

### High rate of fat gain and early development of insulin resistance as predictive markers for decreased lifespan

When correlating markers of energy and glucose homeostasis with lifespan, inclusion of TG mice provides the opportunity to broaden the range of phenotypic variation and to confirm data obtained in WT in a mouse line with known alterations in energy and glucose homeostasis. In TG mice, body weight at the start of the feeding trial was negatively correlated with lifespan, which suggests that the ability of TG to resist deleterious diet effects is strongly linked to the early manifestation and penetration of the transgenic phenotype with regard to body mass effects.

In WT as well as in TG mice, body fat at 24 weeks of age was a better predictor of lifespan than maximum body fat, which was still the case when both genotypes were combined. This suggests that the maximum adiposity reached throughout life is not the most detrimental factor, but rather an early, rapid accretion of body fat after the dietary switch. This is supported by the fact that the rate of body fat gain was one of the strongest predictors, explaining over 30% of the variation in life expectancy.

Correlations per se are not necessarily indicative of a direct causal relationship. However, the observed covariations of the described biomarkers with individual lifespan suggest very strongly that deleterious effects of a high-fat diet on aging and longevity are because of an early rapid body fat accumulation which in turn leads to increased insulin resistance. This is manifested by early increases in blood glucose, which are compensated later on by a continuously increased insulin secretion. These conclusions are strongly supported by the results obtained in the TG mice which showed overall increased insulin sensitivity, and in particular a delayed development of insulin resistance on the high-fat diet resulting in increased longevity on these diets.

As mentioned before, the insulin/IGF1 pathway is thought to play a prominent role in aging and life expectancy. However, studies on different long-lived mouse models suggest that the role of insulin in longevity is very complex. Several mutants such as the Ames and Snell dwarf mice (Dominici *et al.*, 2002; Flurkey *et al.*, 2002) or transgenic mouse models like the S6K knockout mouse (Selman *et al.*, 2009) show extended lifespan as well as increased insulin sensitivity. The GH and IGF1 system have complex feedback mechanisms that, in general, promote insulin resistance (Dominici *et al.*, 2005). Masternak *et al.* (2010) could show that in normal, Ames dwarf, and GH receptor knockout mice, changes in whole body insulin sensitivity were associated with lifespan alterations. They conclude that enhanced insulin sensitivity is a potential marker of increased life expectancy. On the other hand, several reports have shown an increased lifespan in relatively insulin resistant mouse lines such as female IRS-1 null mice (Selman *et al.*, 2008) and brain-specific IRS-2 null mice (Taguchi *et al.*, 2007). In our study, early development of insulin resistance (HOMA-IR week 24) explained only about 10% of the variability in life expectancy of WT and TG together, suggesting that other, possibly independent factors must be involved in determination of lifespan.

So far, the molecular mechanisms leading to the increased survival of HSA-UCP1 mice on the high-fat diets are still not known. It could be linked to a down regulation of ribosomal S6 protein kinase signaling, which was reported to regulate mammalian lifespan supposedly by increasing activity of AMP activated protein kinase (AMPK) (Selman *et al.*, 2009), a master regulator of cellular energy homeostasis (Hardie, 2008). Skeletal muscle mitochondrial uncoupling activates AMPK (Gates *et al.*, 2007; Neschen *et al.*, 2008), and, in turn, AMPK has been shown to regulate energy metabolism by modulating the activity of the histone/protein deacetylase SIRT1 (Canto *et al.*, 2009), a molecule supposed to be involved in gene expression changes that mediate the increase in longevity induced by caloric restriction (Ruderman *et al.*, 2010). One downstream target of AMPK is the mammalian target of rapamycin (mTOR), an important regulator of growth control and metabolism, which is inhibited by AMPK (Kimball, 2006; Shaw, 2009). mTOR has been found to be over-expressed in obesity-linked insulin resistance in mice (Khamzina *et al.*, 2005; Tremblay *et al.*, 2007) and to be upregulated by chronic lipid availability in rats, which could be reversed by exercise training leading to AMPK activation (Rivas *et al.*, 2009). Chronic nutrient excess thus seems to activate mTOR-S6 signaling, which could be counteracted by increased AMPK activity in TG mice. We have previously shown increased skeletal muscle AMPK activation in HSA-UCP1 mice fed a standard chow diet (Neschen *et al.*, 2008). It remains to be investigated if this upregulation of AMPK activity is still evident after long-term feeding of the high-fat diets used here and if it affects mTOR signaling.

Interestingly, it has been shown in *Caenorhabditis elegans* that glucose restriction increased lifespan by inducing mitochondrial respiration and increasing oxidative stress (Schulz *et al.*, 2007), leading to the development of the concept of mitohormesis (Ristow & Zarse, 2010). This concept suggests that increased formation of reactive oxygen species causes an adaptive response by increasing oxidative defense, and thereby ultimately leading to reduced oxidative stress. This provides a common mechanistic denominator for the positive effects of different metabolic interventions on lifespan such as calorie restriction, glucose restriction, and physical activity (Ristow & Zarse, 2010). We have shown a decreased ROS production in isolated muscle mitochondria of HSA-UCP1 mice *in vitro* (Keipert *et al.*, 2010), and preliminary data are indicative of decreased oxidative stress in muscle of TG mice even on high-fat feeding (S. Keipert, A. Voigt, unpublished data), suggesting that decreased oxidative stress could also be a mechanism by which skeletal muscle mitochondrial uncoupling affects aging and longevity.

It remains to be established whether one or several of the described pathways are involved in the regulation of lifespan in our model of combining dietary and metabolic intervention.

## Conclusions

Altogether our study suggests that harmful effects of high-fat diets on lifespan are predominantly linked to early and rapid increases in body fat content, which then lead to early development of insulin resistance. A reduction in dietary carbohydrate in favor of protein ameliorates the damaging effects of high dietary fat exposure by reducing body fat accretion and thus blunting early increases in glucose levels, thereby delaying the development of insulin resistance and hyper-insulinemia. Even without the development of manifest type 2 diabetes, chronically elevated insulin levels and insulin resistance are linked to a decreased lifespan and represent a better predictive marker for reduced longevity than glucose levels alone. Furthermore, a genetic manipulation, which leads to increased weight-specific energy expenditure, reduced body weight, and increased insulin sensitivity, was able to almost completely alleviate the detrimental effects of high-fat diets apparently not by preventing but rather by decelerating the rate of body fat accretion and thus retaining insulin sensitivity and reduced insulin levels over a longer time period. This strong influence of energy metabolism on longevity – especially under unfavorable dietary conditions – suggests that behavioral or pharmacological interventions that target energy metabolism could be able to counteract detrimental long-term dietary effects on aging and longevity.

## Experimental procedures

### Animal maintenance and longevity study

HSA-mUCP1 animals were generated as previously described (Klaus *et al.*, 2005). Experiments were performed with male and female heterozygous HSA-mUCP1 and wild-type (WT) controls maintained on a mixed C57BL/6 – CBA background. Mice were housed in groups of same sex littermates with ad libitum access to food and water at a temperature of 22°C. Animal maintenance and experiments were approved by the animal welfare committee of the Ministry of Agriculture and Environment (State of Brandenburg, Germany).

### Diets

At 12 weeks of age, mice were switched from standard chow diet to one of three semisynthetic diets with different macronutrient composition (Table 5). As a control diet a low-fat high-carbohydrate diet was used (HCLF). The two high-fat diets (HCHF, LCHF) had the same fat content but differed in their protein:carbohydrate ratio. The high-carbohydrate/high-fat diet (HCHF) was matched in carbohydrate content, and the low-carbohydrate/high-fat diet (LCHF) was matched in protein content to the control diet to analyze macronutrient effects.

**Table 5:** Composition of the different semisynthetic diets*

	Control high-carbohydrate/low-fat	High-carbohydrate/high-fat	Low-carbohydrate/high-fat
Casein (g kg^−1^)	410	180	500
Wheat starch (g kg^−1^)	370	430	80
Saccharose (g kg^−1^)	50	50	50
Palm kernel fat (g kg^−1^)	50	180	180
Thistle oil (g kg^−1^)	10	10	10
Linseed Oil (g kg^−1^)	10	10	10
Cellulose (g kg^−1^)	30	70	100
Mineral mixture (g kg^−1^)	50	50	50
Vitamin Mixture (g kg^−1^)	20	20	20
Metabolizable energy (kJ g^−1^)	15.5	17.7	17.5
Protein (energy %)	41.7	16.0	45.0
CHO (energy %)	41.1	41.0	11.5
Fat (energy %)	17.2	43.0	43.6

* Diet components as described (Daenzer *et al.*, 2002).

### Analyses of mouse survival

Survival was assessed from 17 to 23 female mice per group and 18–26 male mice per group. All animals are dead by the time of this report. Between 3 and 9 months of age, we measured a series of parameters relating to individual aspects of energy balance and glucose homeostasis as described above. Mice were monitored daily until they died or were euthanized when they were ill and it was judged that they would not survive for several more days. This time point was entered in the survival statistics. Following criteria for euthanasia were applied: severe weight loss over a period of several days, body fat levels below inability to eat or drink, extreme lethargy (i.e. reluctance to move upon gentle prodding), profound bleeding from open sores or tumors. A higher percentage of mice had to be euthanized on the two high-fat diets apparently because of the increased incidences of scratching-related sores and rapid weight loss. However, there was no apparent genotype effect on this percentage. In order not to stress the animals too much, they were divided into two groups subjected to different measurements as described below. We found no differences in survival between the two treatment groups, suggesting that the measurements had no influence on lifespan.

### Body composition

Body weight and composition were determined monthly using quantitative magnetic resonance (QMR) (Bruker's Minispec MQ10, Housten, TX, USA), a noninvasive method to measure body composition (Tinsley *et al.*, 2004).

### Glucose and energy metabolism

The genotypes on the different diets were divided into two groups of 7–12 animals per gender. In the first group, blood glucose was determined (3 h after food withdrawal) every 12 weeks in whole blood from the tail using a common glucose sensor (Bayer, Germany) and plasma insulin by an ultra-sensitive ELISA assay (DRG Instruments GmbH, Germany). Plasma insulin levels of several transgenic mice were at or below the detection threshold of 0.175 μg L^−1^ at 12 weeks of age. In these cases, 0.175 μg L^−1^ was entered for calculation of mean levels for figure presentation (Fig. 5C,D) but values were not included in statistical calculations. After 48 weeks of dietary intervention (week 60), an intra-peritoneal (i.p.) glucose tolerance test (GTT) was performed. Glucose (2 mg g^−1^ body weight, 20% solution) was applied i.p. three hours after food withdrawal. Insulin levels were measured before, 15, and 30 min after glucose application. Homeostasis Model Assessment for insulin resistance (HOMA-IR) was calculated as described by Lansang *et al.* (2001) using the following function: fasting plasma glucose [mm] × fasting plasma insulin [pm]/22.5.

In the second group, energy expenditure of single mice was measured by indirect calorimetry over 24 h using an open respirometric system after 19 weeks of dietary intervention (week 31 of age), as described before (Katterle *et al.*, 2008). Mice were kept in normal housing cages and had free access to food and water during the measurement. After indirect calorimetry measurements, spontaneous physical activity was assessed in the same cages over a period of 4 days using an infrared method (TSE Systems GmbH, Germany).

### Statistical analyses

Statistical analyses were performed using Stat Graph Prism 5.0 (La Jolla, CA, USA). Kaplan–Meier survival curves were constructed using known birth and death dates with differences between groups evaluated using the log-rank (Mantel-Cox) test. Data were tested for normal distribution and one-way or two-way anova was used to evaluate differences between groups with appropriate *post hoc* tests for normally distributed data. Not normally distributed data were analyzed using a nonparametric test (Mann–Whitney test). Statistical significance was assumed at *P* < 0.05. Correlations (Spearman) were calculated between phenotypic markers and lifespan. Two-tailed *P* values were computed. Data are reported as mean ± SEM unless otherwise indicated.

## Supporting information

**Fig. S1** Kaplan–Meier survival curves of WT and TG (HSA-UCP1) mice fed three different semisynthetic macronutrient diets ad libitum from 12 weeks of age.

**Table S1** Survival characteristics (days) of wild-type (WT) and transgenic (HSA-UCP1) mice fed three different semisynthetic macronutrient diets. Data are reported ± SEM where appropriate.

**Table S2** The Area under the curve (AUC) of male and female WT and TG (HSA-UCP1) mice for the Glucose Tolerance Test (GTT) in week 60 and the lifelong development of blood glucose and plasma insulin levels (data are shown as mean ± SEM).

As a service to our authors and readers, this journal provides supporting information supplied by the authors. Such materials are peer-reviewed and may be re-organized for online delivery, but are not copy-edited or typeset. Technical support issues arising from supporting information (other than missing files) should be addressed to the authors.
