# Supplementary material for: Dietary effects on body composition, glucose metabolism, and longevity are modulated by skeletal muscle mitochondrial uncoupling in mice
Source: Aging Cell. 2011 Feb;10(1):122–36. doi: 10.1111/j.1474-9726.2010.00648.x (PMC3042149; doi:10.1111/j.1474-9726.2010.00648.x)
Supplement: Supplementary file 1 [file acel0010-0122-SD1.doc]

Supporting online material for:

**Dietary effects on body composition, glucose metabolism and longevity are modulated by skeletal muscle mitochondrial uncoupling in mice.** Susanne Keipert, Anja Voigt, Susanne Klaus

**Table S1:** Survival characteristics (days) of wildtype (WT) and transgenic (HSA-UCP1) mice fed three different semi-synthetic macronutrient diets. Data are reported +/- SEM where appropriate.

| **diet** | **median** | **mean** | **range** | **n** | |
| --- | --- | --- | --- | --- | --- |
| **WT male** | | | | |  |
| **HCLF** | 797.5 | 793 +/- 41 | 423-1167 | 20 | |
| **LCHF** | 738 | 634 +/- 53 | 263-976 | 22 | |
| **HCHF** | 593.5 | 589 +/- 41 | 328-965 | 20 | |
| **WT female** | | | | |  |
| **HCLF** | 825 | 823 +/- 32 | 502-987 | 17 | |
| **LCHF** | 754 | 706 +/- 37 | 367-898 | 19 | |
| **HCHF** | 540 | 523 +/- 23 | 273-700 | 22 | |
| **HSA-UCP1 male** | | | | |  |
| **HCLF** | 844 | 839 +/- 35 | 505-1084 | 19 | |
| **LCHF** | 839 | 788 +/- 35 | 315-1303 | 27 | |
| **HCHF** | 785.5 | 748 +/- 35 | 350-932 | 20 | |
| **HSA-UCP1 female** | | | | |  |
| **HCLF** | 871 | 871 +/- 39 | 421-1192 | 23 | |
| **LCHF** | 880 | 846 +/- 40 | 400-1110 | 23 | |
| **HCHF** | 777.5 | 736 +/- 52 | 227-1059 | 20 | |

**Fig.S1** Kaplan Meier survival curves of WT and TG (HSA-UCP1) mice fed three different semi-synthetic macronutrient diets ad libitum from 12 weeks of age. A significant reduction in lifespan of WT HCHF fed mice compared to HCLF fed mice was observed in (**A**) males (p=0.002), and (**B**) in females (p<0.0001, log-rank test). Lifespan of LCHF fed WT mice was significantly different from both other diets only in female mice (**B**). Only a slightly difference of survival curves in (**C**) male tg animals was observed (HCHF vs HCLF, p=0.015, log-rank test) and no differences in female tg mice (**D**)

**Table S2:** The Area under the curve (AUC) of male and female WT and TG (HSA-UCP1) mice for the Glucose Tolerance Test (GTT) in week 60 and the life long development of blood glucose and plasma insulin levels (data are shown as mean ± SEM). Different superscript letters denote significant differences between the diet groups within one genotype (ANOVA).

|  | **WT** | | | | | **HSA-UCP1 (TG)** | | | | | **ANOVA** | |
| --- | --- | --- | --- | --- | --- | --- | --- | --- | --- | --- | --- | --- |
|  | **HCLF** | | **HCHF** | | **LCHF** | **HCLF** | **HCHF** | | **LCHF** | | **diet** | **genotype** |
| **Glucose tolerance test** |  | | | | |  | | | | |  |  |
| AUC/100 Glucose (male) | 24±3 | 23±2 | | 33±4 | | 22±2 | | 22±2 | | 19±1 | ns | 0.0055 |
| AUC/10 Insulin (male) | 28±7a | 70±10b | | 33±4a | | 6±1a | | 25±5b | | 11±2a | >0.0001 | >0.0001 |
| AUC/100 Glucose (female) | 24±2 | 20±3 | | 28±4 | | 19±2 | | 21±2 | | 19±2 | ns | 0.026 |
| AUC/10 Insulin (female) | 16±5 | 45±14 | | 20±3 | | 6±2 | | 14±5 | | 5±0.7 | 0.008 | 0.0004 |
| **Life long development of blood glucose levels** |  | | | | |  | | | | |  | |
| AUC/100 (male) | 55±2 | 60±2 | | 57±3 | | 51±2^ab^ | | 57±3^a^ | | 46±3^b^ | 0.025 | 0.002 |
| AUC/100 (female) | 50±2 | 52±2 | | 51±2 | | 45±1 | | 48±2 | | 44±2 | ns | 0.003 |
| **Life long development of plasma insulin levels** |  |  | |  | |  | |  | |  |  |  |
| AUC/10 (male) | 29±4^a^ | 50±3^b^ | | 34±6^ab^ | | 7±1^a^ | | 19±3^b^ | | 8±1^a^ | >0.0001 | >0.0001 |
| AUC/10 (female) | 11±3^a^ | 37±13^b^ | | 12±1^a^ | | 4±0.8 | | 7±2 | | 3±0.5 | 0,0002 | >0.0001 |
